# Supplementary material for: Parental attitudes to randomised controlled trials in primary dental care: A qualitative study
Source: PLoS One. 2026 Mar 12;21(3):e0330055. doi: 10.1371/journal.pone.0330055 (PMC12981450; doi:10.1371/journal.pone.0330055)
Supplement: S1 File — (DOCX) [file pone.0330055.s001.docx]

# Supplement: Qualitative Interview Topic Guide

*Start recording*

1. Opening

**(Establish Rapport)** [shake hands] My name is Heather and as a dentist and PhD student who has had some involvement with FiCTION, I thought it would be a good idea to interview you, so that I can better inform further dental research.

**(Purpose)** I would like to ask some questions about some dental experiences you’ve had and how much you understand about the FiCTION Trial & my PhD Study (IMPACT) n order to learn more about you and share this information with the rest of the dental team.

**(Motivation)** I hope to use this information to help dental research easier and the patient journey more enjoyable/

**(Time Line)** The interview should take about 60 minutes. Are you available to respond to some questions at this time?

(**Transition:** Let me begin by asking you some questions about your dental health)

II. Body

1. General thoughts
2. (Tell me) What do you understand having good oral health or good dental health to mean?
3. What do you envisage somebody with good oral health to have in terms of teeth or gums?
4. What do you think enables someone to have good dental health?
5. What problems do you think that someone with poor oral health or poor dental health might have?
6. How important is having good dental health to you? Other people? Why do you think it’s important?
7. Previous dental history
8. How often did you go to the dentist when you were a child?
9. Did you have many problems with your teeth as a child?
10. Do you remember going to the dentist as a child as being a good or bad experience?
11. Did you always see the same dentist when you were younger?
    - Were they particularly influential over you at that time?
    - Do you remember them teaching you how to brush your teeth and things?
    - Or was it more to just go and the teeth fixed?
12. Current dental history
13. How often do you go to the dentist now?
14. How important do you think regular visits to the dentist are?
15. What do you do to keep your teeth clean?
16. Where do you think the knowledge to do that came from?
17. Do you go to the same dentist as your child(ren)? If different, why?
18. Research – general thoughts
19. What do you understand by the term research?
20. (Explain research if necessary). Have you take part in any research before? If yes, were there any benefits you felt in taking part?
21. Why do you think dental practices are getting involved in research?
22. There is a big push for the general public to be involved in research now. This would involve primary care settings (e.g. routine doctors, dentists, opticians). Do you think the majority of people would be interested in this? Are there any groups that you don’t think would be willing to take part it in?
23. Would you choose to go to a dental practice that is involved in research or one that wasn’t? Why?
24. How do you feel about children taking part in research?
25. Research – opinions specific to IMPACT/FiCTION
26. How did you feel about being invited to take part in IMPACT? Were you happy to be invited via post, or would you have preferred another option/alternative?
27. What made you decide to take part in IMPACT?
28. Was there anything that worried you about taking part in IMPACT? Has this worry been founded?
29. What did you think about the written information for IMPACT?
30. Did you discuss IMPACT with anybody else before making your decision to take part?
31. IMPACT is a small study that ties in with the FiCTION trial. What did you understand the FiCTION trial is looking at?
32. The FiCTION trial is a randomised controlled trial. Do you know what this means?
33. (Explain randomisation if necessary). How do you feel about a computer picking a treatment arm for your child rather than your dentist/therapist/etc?
34. What do you think your options would be if your child was selected a trial arm that you that you later weren’t happy with? How would you feel about that?
35. Have you found any positives in taking part in IMPACT? What about negatives?
36. Have you found any positives in taking part in FiCTION? What about negatives?
37. Would you participate to take part in another dental study if asked now? Why?

(**Transition:** Well, it has been a pleasure finding out more about you. Let me briefly summarize the information that I have recorded during our interview).

**III Closing**

1. (Summarize). You are very involved in____. You thought_____
2. (Maintain Rapport) I appreciate the time you took for this interview. IS there anything else you think would be helpful for me to know?
3. (Action to be taken) I should have all the information I need. Would it be alright to call you if I have any more questions? Thanks again.

*End recording.*
