# Supplementary material for: Parental attitudes to randomised controlled trials in primary dental care: A qualitative study
Source: PLoS One. 2026 Mar 12;21(3):e0330055. doi: 10.1371/journal.pone.0330055 (PMC12981450; doi:10.1371/journal.pone.0330055)
Supplement: Attachment — Submitted filename: Response to Reviewers.docx [file pone.0330055.s003.docx]

Emily Chenette

Editor-in-Chief

PLOS One

15^th^ January 2026

Dear Editor-in-Chief,

**Re: Parental attitudes to randomised controlled trials in primary dental care: A qualitative study**

Thank you for the comments raised by the academic editor and reviewers. In response to these comments, I write to confirm that the manuscript has been revised to comply with the recommended revisions and changes have been made to the enclosed documents:

| **Document** | **Version** |
| --- | --- |
| Revised Manuscript with Track Changes | Revisions December 2025 |
| Manuscript | Revisions December 2025 |
| Fig 1 TIF | Revisions December 2025 |

In reference to the recommended revisions and edits, I refer to the “Revised Manuscript with Track Changes” document. My responses are given below:

**Academic Editor**

1. **Please ensure that your manuscript meets** **PLOS ONE's style requirements, including those for file naming.**

*Response:* The manuscript has been amended in accordance with PLOS ONE’s requirements including file naming.

2. **Thank you for stating the following in your manuscript:**

**[This research was funded by the Centre for Oral Health Research at Newcastle University and NHS Education for Scotland (through the Scottish Dental Practice Based Research Network).]**

**We note that you have provided funding information that is currently declared in your Funding Statement. However, funding information should not appear in the Acknowledgments section or other areas of your manuscript. We will only publish funding information present in the Funding Statement section of the online submission form.**

**Please remove any funding-related text from the manuscript and let us know how you would like to update your Funding Statement. Currently, your Funding Statement reads as follows:**

**[This research was funded by the Centre for Oral Health Research at Newcastle University and NHS Education for Scotland (through the Scottish Dental Practice Based Research Network). The funders did not play any role in the study design, data collection and analysis, decision to publish, or preparation of the manuscript.]**

*Response:* The funding-related text has been removed from the manuscript. The funding information present in the Funding Statement does not require amendment.

3. **We note that this data set consists of interview transcripts. Can you please confirm that all participants gave consent for interview transcript to be published?**

**If they DID provide consent for these transcripts to be published, please also confirm that the transcripts do not contain any potentially identifying information (or let us know if the participants consented to having their personal details published and made publicly available). We consider the following details to be identifying information:**

**- Names, nicknames, and initials**

**- Age more specific than round numbers**

**- GPS coordinates, physical addresses, IP addresses, email addresses**

**- Information in small sample sizes (e.g. 40 students from X class in X year at X university)**

**- Specific dates (e.g. visit dates, interview dates)**

**- ID numbers**

**Or, if the participants DID NOT provide consent for these transcripts to be published:**

**- Provide a de-identified version of the data or excerpts of interview responses**

**- Provide information regarding how these transcripts can be accessed by researchers who meet the criteria for access to confidential data, including:**

**a) the grounds for restriction**

**b) the name of the ethics committee, Institutional Review Board, or third-party organization that is imposing sharing restrictions on the data**

**c) a non-author, institutional point of contact that is able to field data access queries, in the interest of maintaining long-term data accessibility.**

**d) Any relevant data set names, URLs, DOIs, etc. that an independent researcher would need in order to request your minimal data set.**

*Response:* Participants did not provide consent for the transcripts to be published. Consent was obtained for de-identified interview responses to be used and these have been inserted within the manuscript. The transcripts have been uploaded in a Newcastle University storage repository (<https://data.ncl.ac.uk/>) but they are not openly available due to consent considerations and given the sensitive nature of the research topic. Transcripts can only be unlocked and accessed by researchers who met the criteria for access to this confidential data; further data access advice/ queries can be sought from [fmsethics@ncl.ac.uk](mailto:fmsethics@ncl.ac.uk). The URL is: <https://figshare.com/s/c05f754f3cd27d3e40ee>. The NHS Health Research Authority NRES Committee North East - Newcastle & North Tyneside 1 Research Committee is responsible for imposing restrictions on the data. The REC reference number is 13/NE/0180. The data is not owned by a third party.

**Reviewer 1**

1. **Abstract – I would recommend that the authors spell out “NHS” in full at its first mention before using the abbreviation to ensure clarity for all readers.**

*Response*: The manuscript has been amended to include the meaning of the abbreviation “NHS” at first use and now states “National Health Service (NHS)”. This affects:

- Page 2, Line number 25.
- Page 5, Line number 89.

2. **Introduction - I recommend that the authors spell out “NIHR” in full at its first mention before using the abbreviation to ensure clarity for all readers.**

*Response*: The manuscript has been amended to include the meaning of the abbreviation “NIHR” at first use and now states “National Institute for Health Research (NIHR)”. This affects:

- Page 4, Line number 61.

3. **Introduction - The introduction would benefit from stating potential barriers related to parents not fully understanding the importance of research, and specifically RCTs, in the context of paediatric dental care.**

*Response:* The manuscript has been amended to include the importance of having a representative research population within dental research to minimise dental health disparities. Text has been added to the introduction section on Page 4, Line numbers 78 – 87.

This new paragraph reads *“Learning from parents who decline their child from participating in dental research is critical if we aim to have a more representative research population and subsequently a more valid and generalisable evidence base. Dental research has shown that children living in the most deprived areas of the country are more than twice as likely to have experienced dentinal decay (32.2%) as those living in the least deprived areas (13.6%) [13]. There is also evidence of ethnic inequalities in oral health within the UK, largely based on limited data from England [14]. Lower participation of under-represented groups threatens to exacerbate existing paediatric dental health disparities. Understanding potential barriers related to parents not fully understanding the importance of dental research in the context of paediatric dental care needs to be more robust to help shape policies to address these inequalities. “*

4. **Methods – In the data analysis section, the presentation of multiple analytic approaches (essentialist, contextualist, constructionist thematic analysis, framework method) makes the process difficult to follow. It would be helpful for the authors to clarify which approach was primarily adopted, and how the different methods were integrated or distinguished.**

*Response:* The manuscript has been amended for clarity. These methods are all used under the umbrella of thematic analysis. Text has therefore been added to the Data analysis section on Pages 14 and 15, Line numbers 284-300.

The revised paragraph now reads *“The methodological orientation used to underpin this study was thematic analysis [30]. An essentialist method was used initially when exploring parents’ dental experiences to focus on what they felt truly mattered to them. A constructionist method which supported parents to reflect and make sense of their lived experiences was then employed when discussing their decisions regarding their children’s dental journey and entry into screening for the FiCTION RCT [30]. A contextualist method was then used with parents by the researchers to check their understanding of the information the parent had given, to enable further discussion and allow clarification. These methods are all used under the umbrella of thematic analysis. Thematic analysis was selected as it can both reflect reality (experiences, meanings and the reality participants have reported), and unpick the surface of “reality” (the ways individuals have made a meaning of their experiences) [30]. Thematic analysis has been suggested as suitable for those early in their qualitative research carer. NVivo [29] was used as a tool to manage the transcripts and to select and label sections of dialogue, thereby creating a method of indexing. Sections were highlighted and grouped together into electronic files. The data were then collated into potential themes which enabled HC, as the data analyst, to become familiar with the data collected. The Framework Method of qualitative analysis [31] was then applied to the dataset [32] as a method to organise and manage the dataset”*.

5. **Methods - It would be useful to include a participant flow chart for the two study locations. For example, indicating the number of eligible parents at each site, the number who completed the baseline questionnaire, the number of parents contacted, and the number of parents interviewed. This addition would enhance transparency regarding recruitment across sites.**

*Response*: Text has been added to the Methods Design section on Pages 6-8, Lines 147-186. Two tables (Table 1 & 2) have also been included.

The new paragraphs now read:

*“Of the 42 dental practices contacted, 13/26 (50%) FiCTION practices in Scotland and 14/16 (88%) FiCTION practices in north-east England agreed to take part in the research project. In total, across all 27 dental practices, 2980 parents, slightly fewer than expected, were identified as being potentially eligible for the research project and subsequently sent invitation packs and asked to return a completed consent form, they were only included if they opted into the research project. In total, 332 parents (11.1%) of those sent the research project invitation pack returned the consent form. Of these parents, the child’s FiCTION status was ascertained as; Child participating in FiCTION (n=66), Child not participating in FiCTION as family declined (n=6) and Child not participating in FiCTION as not eligible (n=258). As planned, the six parents whose children were not participating in FiCTION, as the family had declined, were combined with the parents whose children were not participating in FiCTION, because they were not eligible, to produce a single Non-FiCTION group (n=264).*

*Almost all of the 332 parents (n=312, 94.0% of those returning a consent form, 10.5% of those contacted) who returned the consent form agreed to participate in the research project. The rate of consent was very similar irrespective of the child’s FiCTION status. All consenting parents met the research project criteria and were then sent a baseline questionnaire. In total, and following the issue of reminder questionnaires to those who did not respond to the initial mailing, 261 parents (84% of those consenting to the research project) returned a completed baseline questionnaire and were included in the study (Table 1). It can be seen, by comparing the country of residence, that more parents were recruited from England than Scotland, irrespective of FiCTION status (overall 65.1% England, 34.9% Scotland); however, this was proportional to the number of invitation packs sent in England and Scotland. There was the same ratio of participants from England and Scotland in both the FiCTION and Non-FiCTION status groups.*

***Table 1. Number of questionnaires returned by FiCTION and Non-FiCTION parents at baseline and after 18 months, in Scotland and England.***

|  | ***FiCTION*** | | ***Non-FiCTION*** | | ***Total*** | |
| --- | --- | --- | --- | --- | --- | --- |
|  | ***Baseline*** | ***18 months from baseline*** | ***Baseline*** | ***18 months from baseline*** | ***Baseline*** | ***18 months from baseline*** |
|  | ***n*** | ***n*** | ***n*** | ***n*** | ***n*** | ***n*** |
| ***Scotland*** | *19* | *16* | *72* | *58* | *91* | *74* |
| ***England*** | *36* | *29* | *134* | *97* | *170* | *126* |
| ***Total*** | *55* | *45* | *206* | *155* | *261* | *200* |

*The baseline study sample was 261 parents residing in the UK; 55 FiCTION parents and 206 Non-FiCTION parents. The mean age of parents was 38.1 years (SD 5.8 years), and 54% (140/261) were in the age group 36-45 years. There was little baseline difference between parents in the FiCTION and Non-FiCTION groups in terms of their age, relationship to the child or ethnicity (Table 2). The majority of questionnaires, irrespective of FiCTION status, were completed by mothers and by parents who identified themselves as white. There were large differences in the highest education level completed between the FiCTION and Non-FiCTION groups, with higher levels of educational attainment amongst the Non-FiCTION group (29.5% Non-FiCTION completed postgraduate education versus 16% FiCTION), suggesting either a different demographic mix of parents, a differing understanding of terminology used to describe education attainment or simply chance variation.*

***Table 2. Demographic characteristics of FiCTION and Non-FiCTION parents at baseline.***

| ***Baseline characteristics*** | | | | | | | |
| --- | --- | --- | --- | --- | --- | --- | --- |
|  | ***FiCTION***  ***(n= 55)*** | | ***Non-FiCTION (n=206)*** | | | ***Total***  ***(n=261)*** | |
|  | ***n*** |  | ***n*** |  | | ***n*** |  |
| ***Age (years)*** |  |  |  |  | |  |  |
| *Mean* |  | *38.1* |  | *38.1* | |  | *38.1* |
| *Standard Deviation* |  | *6.7* |  | *5.5* | |  | *5.8* |
| *Total responses* | *47* |  | *197* |  | | *244* |  |
|  |  |  |  |  | |  |  |
| ***Relationship to child*** |  |  |  |  | |  |  |
| *Mother* | *46* | *(86.8%)* | *182* | *(88.3%)* | | *228* | *(88.7%)* |
| *Father* | *7* | *(13.2%)* | *16* | *(7.8%)* | | *23* | *(8.9%)* |
| *Other* | *0* | *(0.0%)* | *6* | *(2.9%)* | | *6* | *(2.3%)* |
| *Total responses* | *53* |  | *204* |  | | *257* |  |
|  |  | |  | | |  | |
| ***Ethnicity*** |  | |  | | |  | |
| *White* | *53* | *(96.4%)* | *197* | *(96.1%)* | | *250* | *(96.2%)* |
| *Indian, Pakistani or Bangladeshi* | *0* | *(0.0%)* | *5* | *(2.4%)* | | *5* | *(1.9%)* |
| *Mixed race* | *0* | *(0.0%)* | *3* | *(1.5%)* | | *3* | *(1.2%)* |
| *Other* | *2* | *(3.6%)* | *0* | *(0.0%)* | | *2* | *(0.8%)* |
| *Total responses* | *55* |  | *205* |  | | *260* |  |
|  |  |  |  | |  |  |  |
| ***Highest level education completed*** |  |  |  | |  |  |  |
| *Primary school* | *4* | *(8.0%)* | *5* | | *(2.5%)* | *9* | *(3.4%)* |
| *Secondary school* | *12* | *(24.0%)* | *13* | | *(6.5%)* | *25* | *(9.6%)* |
| *Some additional training* | *12* | *(24.0%)* | *63* | | *(31.5%)* | *75* | *(28.7%)* |
| *Undergraduate university* | *14* | *(28.0%)* | *60* | | *(30.0%)* | *74* | *(28.4%)* |
| *Postgraduate university* | *8* | *(16.0%)* | *59* | | *(29.5%)* | *67* | *(25.7%)* |
| *Total responses* | *50* |  | *200* | |  | *250* |  |
|  |  |  |  | |  |  |  |
| ***Country of Residence*** |  |  |  | |  |  |  |
| *England* | *36* | *(65.5%)* | *134* | | *(65.0%)* | *170* | *(65.1%)* |
| *Scotland* | *19* | *(34.5%)* | *72* | | *(35.0%)* | *91* | *(34.9%)* |
| Total responses | 55 |  | 206 | |  | 261 |  |

A participant flow chart has been inserted as a Figure (Fig 1. Participant Flow diagram) into the revised manuscript within the results section on Page 15. In accordance with PLOS ONE's style requirements, the participant flow chart has been submitted in a separate document.

6. **Results - In the participant profile table, it would be helpful to provide further detail on the category “other” for parent ethnicity. Clarifying what this category includes would improve transparency and interpretability of the data.**

*Response:* The manuscript has been amended to include further detail on the parents‘ ethnicity. Text has also been added to Table 4 on Page 18, Line number 327.

7. **Results - In the description accompanying the participant profile table, I suggest including the mean age of the parents.**

*Response*: The manuscript has been amended to include the mean age of the parents. Text has been added to the results section on Page 19, Line numbers 329-330. This now reads: *The mean age of parents was 40.4 years (SD 7.3 years), which was slightly higher than the full baseline study sample (n=261) where the mean age of parents was 38.1 years (SD 5.8 years).*

8. **Results - The Results section is at times difficult to follow. For example, under “Dental Health” themes 2 and 3 currently contain relatively little information. It may strengthen the presentation to consider reorganizing or merging these themes in a way that avoids sections with limited content.**

*Response:* Thank you for this recommendation. We have merged themes 2 and 3. The manuscript has been amended on Page 23, Lines 417-419. This now reads *“Theme 2: Poor dental health impacts on nutritional, psychological and social performance but dental practice selection and dental attendance have nothing to do with research.”*

9. **Discussion - The discussion section should be made more concise by focusing on the key takeaways that directly relate to the study’s objectives. Emphasizing how these findings connect to the current literature would strengthen the interpretive value and clarity of the manuscript.**

*Response:* The manuscript has been amended under objectives and the section which focuses on the findings and value of the study.

The objectives of the qualitative study have been listed on Page 6, Lines 118-126.

- The first objective has been referred to on Page 33, Line 664. This now reads “*With respect to the first qualitative study objective, parents’ views regarding their own dental health were varied.”*
- The second objective has been referred to on Page 34, Line 688. This now reads *“With respect to the second qualitative study objective, most FiCTION parents understood the general aspects and advantages of participating in the trial, such as the nature of the study, the potential benefit to other children, the notion of voluntary participation and the possibility of withdrawal at any time.”*

The new paragraph which focuses on the findings and value of the study is on Page 36, Lines 752-766. This now reads:

*“The NHS is in a ‘critical condition’ [45] and some politicians are openly calling for the NHS’ founding principles to be abandoned and the taxpayer-funded, free at the point of need health service, to be scrapped [46]. Satisfaction with NHS dentistry has continued to collapse; as recently as 2019 this was at 60%, but it has now fallen to a record low of 20% [47]. Dissatisfaction levels (55%) are the highest for any specific NHS service asked about [47]. Despite qualitative interviews suggesting that parents value the need for research in primary dental care, the mechanisms for this remain complex and challenging. Primary care dentistry is complex. There is a clear need for better quality evidence on which to base practice and one of the keys to generating this evidence is to conduct primary care dental research studies. The British Dental Association estimates 13 million adults - over 1 in 4 - are struggling to find NHS dental care [48]. Worse, evidence shows that even amongst those who do get appointments, it is patients with the greatest clinical need who miss out [46]. There is a recognised need to encourage patient involvement in research, with a focus on under-represented groups such as young people, Black people and people of South Asian heritage [46]. This paper highlights that further research is required explore the best methods to achieve engagement with patients in primary dental care research.”*

10. **Strengths and weakness of the study - Line 669 to 672: The text in this section appears to be incomplete, with some words or parts of sentences missing. Please review and revise for clarity.**

*Response:* The manuscript has been amended for clarity on Page 37, Line 783. This now reads: *“Interviews were completed when further interviewing generated no additional themes [20].”*

11. **Strengths and weakness of the study - The age of the data set (2015) may affect the relevance of the findings. Consider discussing any changes in context since 2015 that might influence interpretation or applicability.**

*Response:* The manuscript has been amended to include further detail on the interpretation of the data in light of the age of the data set. Text has been added to Page 38, Line numbers 805-808. This now reads: *Whilst the data was collected some time ago (2015), satisfaction with NHS dentistry has only worsened [47].* *Indeed the relevance and applicability of the study is even greater since the recognition of the importance of the incorporation of under-represented groups such as young people in research has grown over this time period.*

**Reviewer 2**

1. **Data Availability: Since this is a qualitative study, full data sharing is not recommended due to participant confidentiality and ethical considerations. It will be helpful to clarify this in the Data Availability Statement. A possible way to phrase it is as follows:
   [Qualitative interview transcripts may contain personally identifiable information that cannot be shared with the public. Researchers who meet the criteria for accessing confidential data can access it through the Newcastle University Research Data Service].**

*Response:* The study team are in agreement with the reviewer and will make this change in the online submission form.

2. **Rigor: it is important to outline in the Results or Discussion section when saturation was reached. This minor addition will ensure full adherence to qualitative reporting standards [e.g., saturation was reached after the 15th/18th interview].**

*Response:* The manuscript has been amended for clarity on Page 16, Lines 323-324. This now reads: *“Data collection was stopped after 18 interviews at the point of ‘informational redundancy’ (the point at which the researcher began to hear the same comments and felt the new data was redundant of the data already collected) and it was felt that saturation had been reached after the 18^th^ interview [34].”*

We look forward to hearing from you in due course.

Yours sincerely,

Heather Coventry
